# Quantification of Compatibility Between Polymeric Excipients and Atenolol Using Principal Component Analysis and Hierarchical Cluster Analysis

**DOI:** 10.1208/s12249-021-02143-2

**Published:** 2021-11-19

**Authors:** Barbara Rojek, Maria Gazda, Marek Wesolowski

**Affiliations:** 1 Department of Analytical Chemistry, Medical University of Gdansk, Gen. Hallera 107, 80-416 Gdansk, Poland; 2 Faculty of Applied Physic and Mathematics, Gdansk University of Technology, Narutowicza 11/12, 80-233 Gdansk, Poland

**Keywords:** Polymeric excipients, Atenolol, Compatibility/incompatibility, Thermal and non-thermal methods

## Abstract

An important challenge to overcome in the solid dosage forms technology is the selection of the most biopharmaceutically efficient polymeric excipients. The excipients can be selected, among others, by compatibility studies since incompatibilities between ingredients of the drug formulations adversely affect their bioavailability, stability, efficacy, and safety. Therefore, new, fast, and reliable methods for detecting incompatibility are constantly being sought. Hence, the purpose of this work was to assess the usefulness of a heating, cooling, and reheating differential scanning calorimetry (DSC) program for detecting potential incompatibilities between atenolol, an active pharmaceutical ingredient (API), and polymeric excipients. Hot-stage microscopy (HSM), Fourier transform infrared (FTIR) spectroscopy, and powder X-ray diffraction (PXRD) were used as supporting techniques. Additionally, principal component analysis (PCA) and hierarchical cluster analysis (HCA) served as tools to support the interpretation of the data acquired from the DSC curves and FTIR spectra. As the alterations in the shape of the DSC peak of atenolol which are indicative of incompatibility are visible only on the cooling and reheating curves of the mixtures, the DSC heating–cooling–reheating program was found to be very useful for identifying potential incompatibilities in the binary mixtures of atenolol and polymeric excipients. The melting and recrystallization of atenolol alone and in its mixtures were also confirmed by HSM, while FTIR displayed changes in the spectra of mixtures due to incompatibility. These studies revealed that atenolol is incompatible with hydroxyethylcellulose, hypromellose, and methylcellulose. PXRD measurements at room temperature revealed that the crystallinity of atenolol did not change in these mixtures. However, its crystallinity was reduced in the mixtures previously heated up to 155 °C and then cooled to 25 °C.

**Graphical abstract:**

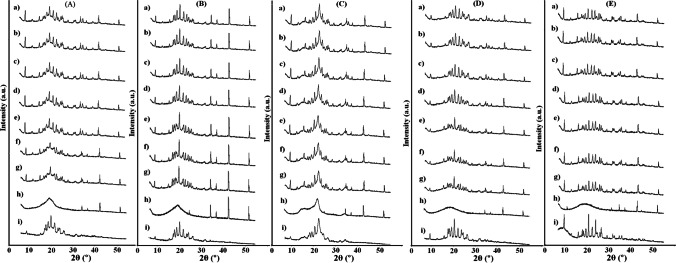

## INTRODUCTION

Compatibility of active pharmaceutical ingredients (APIs) with excipients is an important issue in preformulation studies since it ensures the stability, bioavailability, and manufacturability of solid dosage forms (1). Compatibility means that APIs and excipients can be mixed together without undesired physical or chemical interactions between ingredients. Appearance of the chemical interactions contributes to the reduction of the quantity of API, which is related to its absorption and therapeutic effect. These incompatibilities are the result of the acid-base, hydrolysis, photodegradation, polymerization or decomposition reactions. However, undesired changes in the solubility, dissolution rate, and in consequence the reduction of API bioavailability are the result of physical interactions (1, 2). These incompatibilities can be due to adsorption of drug substance by excipient, drug substance complexation, its amorphization or co-crystallization. Both physical and chemical incompatibilities have destructive effect on the efficacy, safety, stability and quality of the drug product. Thus, it is essential to choose the appropriate excipients by taking into account the physical and chemical properties of APIs (3).

Currently, there is a trend to use natural polysaccharides for this purpose (4). Given the positive qualities of natural polysaccharides such as degradability, nontoxicity, cheapness, abundance, renewability, and sustainability, in addition to their importance for life processes and biological properties, including cell recognition and interactions, enzymatic degradability, and semblance to the extracellular matrix, they are convenient excipients in drug formulation (5). It should also be noted that polysaccharides exhibit great diversity with regard to features, due to variety in the composition of monosaccharide units, link types and patterns, chain length, and shapes. These features affect, among others, solubility, gelling ability, flow behavior, and surface characteristics (3). The primary excipients in the group of natural polysaccharides are starch, modified starch, and cellulose plus its derivatives (6). These polymers are arousing great interest, because they are less variable and have fewer aging issues than some of the traditional excipients (7).

Despite having been defined as inactive or inert ingredients, excipients may nonetheless adversely affect the physical and chemical properties of APIs, causing a reduction in the effectiveness of the dosage form. Therefore, a need exists to check appropriate excipients for specific APIs through the use of sensitive and effective analytical methods. API incompatibility with excipients is commonly detected under accelerated testing conditions and by differential scanning calorimetry (DSC) as screening methods. However, as the results of these tests do not always yield conclusive results, new and more effective methods are constantly being sought (3).

For several decades, the same set of methods has been routinely employed in compatibility studies, including thermal methods such as standard DSC, thermogravimetric analysis (TGA), and non-thermal methods such as FTIR spectroscopy, powder X-ray diffraction (PXRD), or, more rarely, solid-state nuclear magnetic resonance (ssNMR) spectroscopy (1). Recently, multivariate statistical methods have been used to obtain unambiguous conclusions from the uncertain results of thermal and non-thermal methods. Their use is essential because these methods allow us to obtain unequivocal conclusions from experimental data (8, 9).

Standard single heating DSC is a common method in fast API-excipient compatibility studies. An advantageous solution may be to use sample cooling after heating and then reheating. The use of a heating–cooling–reheating program enables additional information to be obtained about the thermal behavior of the ingredients and their mixtures. The cooling cycle provides information on the crystallization temperature and degree of crystallinity of the sample. Second heating then provides information about the new thermal history of the sample. A comparison of the DSC curves from the first and second heating reveals potential differences in these curves. If the DSC curves for the first and second heating are the same, this indicates that the sample remained stable throughout the DSC procedure [10, 11]. Therefore, the purpose of the present work was to assess the usefulness of the heating–cooling–reheating program in detecting potential incompatibilities between atenolol and polymeric excipients. The aim of this work follows from the hypothesis that the heating-cooling-reheating technique would be more efficient in detecting potential incompatibility in atenolol-polymeric excipients combinations, than the traditional thermo-analytical techniques. To obtain reliable conclusions, HSM, FTIR, and PXRD were used as supporting tools, while multivariate statistical methods were applied for improving the interpretation of the DSC and FTIR data. It was also hypothesized that integration of PCA and HCA with non-thermal techniques would increase the propensity of detecting incompatibility between atenolol and polymeric excipients. Atenolol was used as a model drug. This is a β-blocker agent for the treatment of cardiovascular diseases [12], arterial hypertension, ischemic heart disease and cardiac arrhythmias, which along with cancer are the most common cause of human death. Its dosage forms are prepared using different excipients, among others, polymeric excipients. Therefore, polymeric excipients were selected for this study due to their properties useful in the solid dosage drug technology. First of all, polymeric excipients enable controlled release of the drug substance. Thus, the use of polymeric excipients in the solid drug technology provides new possibilities for medicine, including reaching by the drug substance the selected tissues and cells.

## MATERIALS AND METHODS

### Materials

**Fig. 1.**
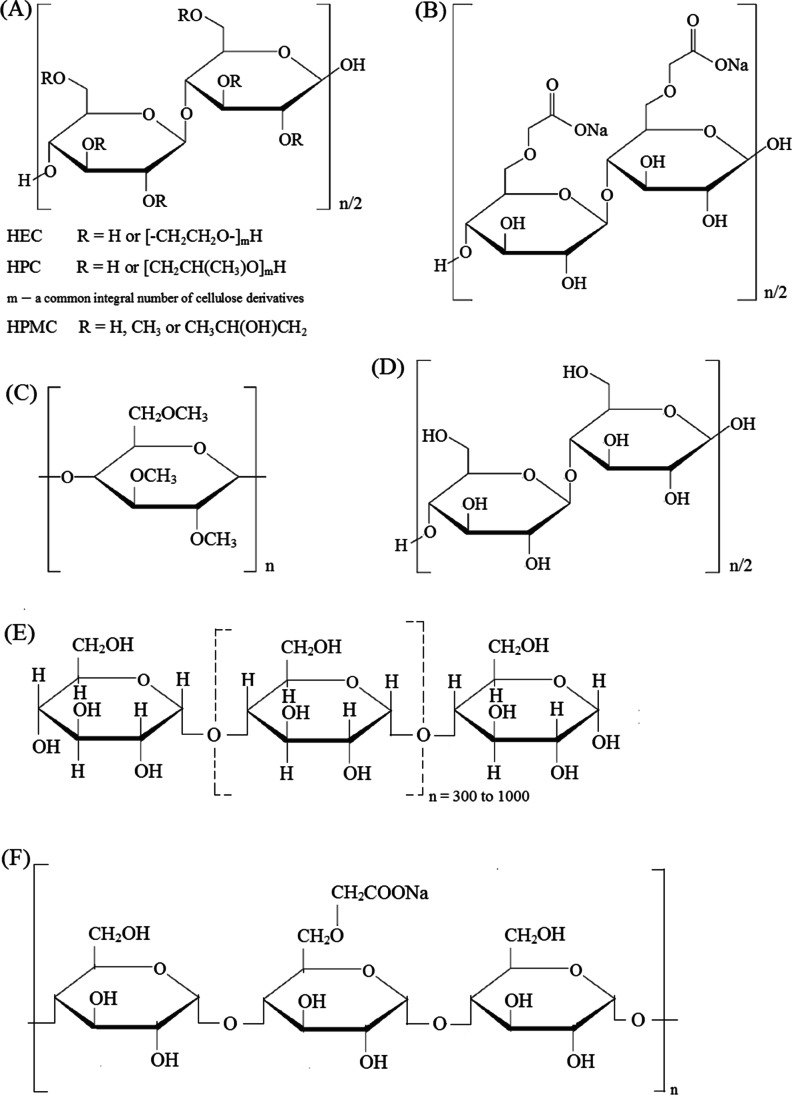
Structural formulas of **A** hydroxyethylcellulose (HEC), hydroxypropylcellulose (HPC), hydroxypropylmethylcellulose (HPMC), **B** sodium carboxymethylcellulose (CMC), **C** methylcellulose (MC), **D** microcrystalline cellulose (MCC), **E** pregelatinized starch (PGS), and **F** sodium starch glycolate (SSG)

**Fig. 2.**
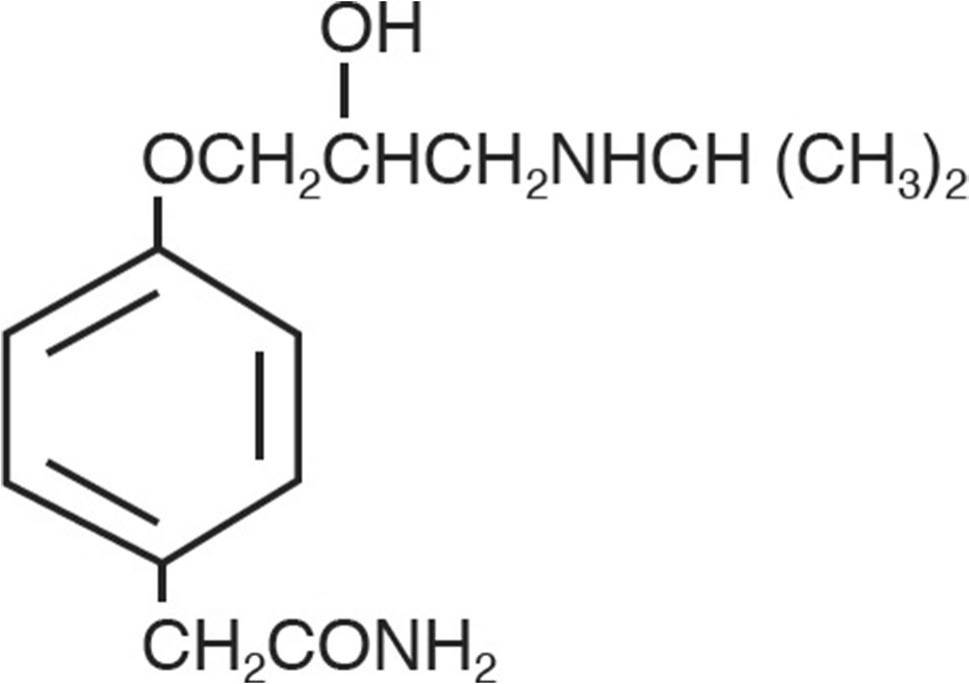
Structural formula of atenolol (At)

The chemical formulas of polymeric excipients are shown in Fig. 1. Hydroxyethylcellulose (HEC), hydroxypropylcellulose (HPC), hydroxypropylmethylcellulose (HPMC, hypromellose), and sodium carboxymethylcellulose (CMC) were provided by Sigma-Aldrich (Steinheim, Germany), methylcellulose (MC) by Shin-Etsu Chemical Co. (Tokyo, Japan), and microcrystalline cellulose (MCC, Avicel PH 101) by FMC Corp. Europe N.V. (Brussels, Belgium). Pregelatinized starch (PGS, Starch 1500) was purchased from Colorcon (Harleysville, PA, USA), sodium starch glycolate (SSG, Viva Srar®) from JRS Pharma (Rosenberg, Germany), and Atenolol (At) (Fig. 2) from Polpharma (Starogard Gdanski, Poland).

Homogeneous binary mixtures consisting of polymeric excipients and atenolol at 50:50 mass ratios were prepared by gently mixing the ingredients for 10 min by pestle in a porcelain mortar. A 50:50 (w/w) physical mixture of atenolol and excipient was chosen to highlight the probability of interaction occurrence. The higher excipient content produce the higher thermal events due to the presence of excipient. Hence, it makes easier detection of potential interactions [13, 14].

#### Thermal Methods

DSC curves of the samples (about 4.20 mg) in aluminum pans with lids with two holes were performed using an 822e differential scanning calorimeter from Mettler Toledo (Schwerzenbach, Switzerland) with STARe software. The tests were carried out in nitrogen (purity 99.9996%) with a flow rate of 70 ml min^−1^. Indium (99.999%) and zinc (99.998%) (both obtained from Mettler Toledo) were used to calibrate the DSC device. The obtained values were as follows: indium (*m.p.* 156.6 °C, *ΔH*_*f*_ = 28.45 J g^-1^) and zinc (*m.p.* 419.6 °C, *ΔH*_*f*_ = 107.50 J g^-1^).

Heating–cooling–reheating measurements were made as follows: heating in the range of 25–200 °C (first heating); isothermal at 200 °C for 2 min; cooling from 200 to − 25 °C (cooling cycle); isothermal at − 25 °C for 2 min; and reheating from − 25 to 200 °C (second heating). All measurements were performed at a 10 °C/min heating rate.

The thermal decomposition of polymeric excipients, atenolol, and their mixtures were carried out using an OD-103 derivatograph (MOM, Budapest, Hungary). The samples (200 mg) were heated in four flat-bottomed platinum pans to 700 °C in air, at a 5 °C min^−1^ heating rate, with alumina being used as a reference sample. The derivatograph was calibrated using calcium oxalate dihydrate.

Hot-stage microscopy (HSM) studies were performed using an Olympus type BX41 thermomicroscope (Shinjuku, Japan) with a SC 30 digital camera and Olympus CellA software. A semic microscope heating table controlled by Heating Desc Shimaden software (Bioelectronics, Cracow) was used for sample heating at a rate of 10 °C min^−1^ in the range of 25–250 °C. The device was calibrated using indomethacin, succinic acid, glutaric acid, and caffeine. The linear relationship between melting points from DSC and HSM with a correlation coefficient of 0.9990 (*y* = 0.804*x* + 3.695) was obtained.

### Fourier Transform Infrared Spectroscopy

FTIR spectra were recorded using a Nicolet 380 FTIR device from Thermo Fisher Scientific (Madison, WI, USA) with a DTGS KBr detector, in the spectral range of 4000–400 cm^−1^ with a resolution of 4 cm^−1^, operating with OMNIC software. The sample spectrum with 16 scans was preceded by background recording. KBr pellets were prepared in an agate mortar by mixing 1 mg sample with 100 mg potassium bromide (Merck, Darmstadt, Germany) and compressing by a Specac hydraulic press (Orpington, England) with a KNF vacuum pump (Neuberger, France).

### Powder X-ray Diffraction

PXRD patterns were collected using the Philips X’pert Pro MPD system with CuKα radiation (1.541 Å). The diffraction patterns were taken over 2θ range of 7-55° with the tube settings of 40 kV and 30 mA. The high-temperature X-ray diffraction (HT-XRD) analyses were performed with Anton Paar HTK system. The patterns were collected in isothermal conditions at selected temperatures between room temperature and 155 °C. The PXRD diffractometer was calibrated using the polycrystalline silicon standard.

### Data Analysis

Principal component analysis (PCA) was used for interpretation of the DSC and FTIR data. The onset and peak temperatures, peak heights and widths, and the enthalpies obtained from the heating, cooling, and reheating DSC curves were included for the PCA calculations. These values were variables, while the objects were polymeric excipients, atenolol, and their mixtures. The matrix for calculations based on FTIR data consisted 17 objects (rows) and 402 variables (columns). In this matrix, atenolol, polymeric excipients and their binary mixtures at 50:50 mass ratio were used as rows, whereas the columns included the absorbance values acquired every 4 cm^-1^ from FTIR spectra of the analyzed samples. For the statistical calculations, the spectral regions of 3600 – 2800 cm^-1^ and 1800 – 1000 cm^-1^ were selected. All data from the raw FTIR spectra were standardized. Covariance matrices formed the basis for PCA calculations.

Hierarchical cluster analysis (HCA) was used to evaluate the similarity between the mixtures and their ingredients using the DSC and FTIR data. Ward’s method was used as the clustering method, Euclidean distance as a distance measure [15], and Sneath’s index criterion to determine the number of significant clusters at 1/3 of the maximum distance [16]. Both PCA and CA calculations were conducted using Statistica 13.3 software (StatSoft Inc., Tulsa, OK, USA).

## RESULTS AND DISCUSSION

### Compatibility Study by Thermal Methods

**Fig. 3.**
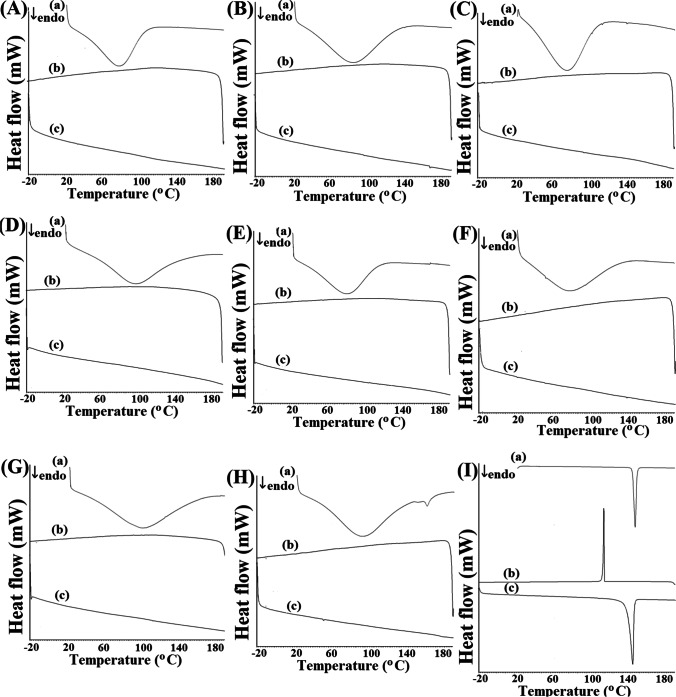
DSC curves for **A** HEC, **B** HPC, **C** HPMC, **D** CMC, **E** MC, **F** MCC, **G** PGS, **H** SSG, and **I** atenolol, where **a** heating, **b** cooling, and **c** reheating

DSC curves of polymeric excipients are presented in Fig. 3, and data on their dehydration is summarized in Table I. The DSC curves show broad endothermic peaks assigned to dehydration with the peak temperature from 79.5 °C for HPMC to 105.8 °C for PGS. TGA curves revealed that decomposition occurs above 200 °C. As shown in Table II, polymeric excipients dehydrate in the temperature range of 25–100 °C or 25–160 °C. The following two steps of mass losses lead to the degradation of polysaccharide molecules. Unlike polysaccharides, atenolol melts at 154.4 °C, as confirmed by a sharp endothermic DSC peak (Fig. 3I); therefore, no mass loss is observed on the TGA curve. Atenolol then decomposes in two steps at the temperature range of 30–580 °C (Table II).

**Table I:** Results of DSC Heating for Polymeric Excipients

Polymeric excipient	*T*_onset_ (°C)	*T*_peak_ (°C)	Δ*H*_dehydration_ (J g^−1^)
HEC	28.65	83.15	192.48
HPC	25.57	88.84	359.42
HPMC	27.89	79.54	121.84
CMC	26.88	101.96	374.82
MC	25.98	84.19	191.22
MCC	26.63	80.25	224.17
PGS	26.36	105.81	320.50
SSG	27.26	95.32	276.84

**Table II:** Results of Thermal Decomposition for Atenolol and Polymeric Excipients

Sample	First step		Second step		Third step	
	Temperature range (°C) (Peak temperature, °C)	Mass loss (%)	Temperature range (°C) (Peak temperature, °C)	Mass loss (%)	Temperature range (°C) (Peak temperature, °C)	Mass loss (%)
At	30–390 (220, 250)	65.0	390–580 (440)	35.0	-	-
HEC	25–100 (40)	6.0	100–350 (230, 275)	78.0	350–500 (400)	16.0
HPC	25–120 (45)	9.0	120–320 (260)	74.0	320–600 (380, 540)	17.0
HPMC	25–120 (35)	5.5	120–320 (280)	79.5	320–500 (420)	15.0
CMC	25–130 (50)	12.0	130–390 (240)	50.0	390–700 (560)	25.0
MC	25–110 (50)	5.0	110–310 (240, 280)	79.0	310–500 (390)	16.0
MCC	25–150 (50)	6.0	150–380 (270)	87.0	380–600 (540)	7.0
PGS	25–160 (50)	11.0	160–330 (250)	72.0	330–460 (420)	17.0
SSG	25–160 (50)	13.5	160–380 (220)	52.5	380–700 (520)	24.0

**Fig. 4.**
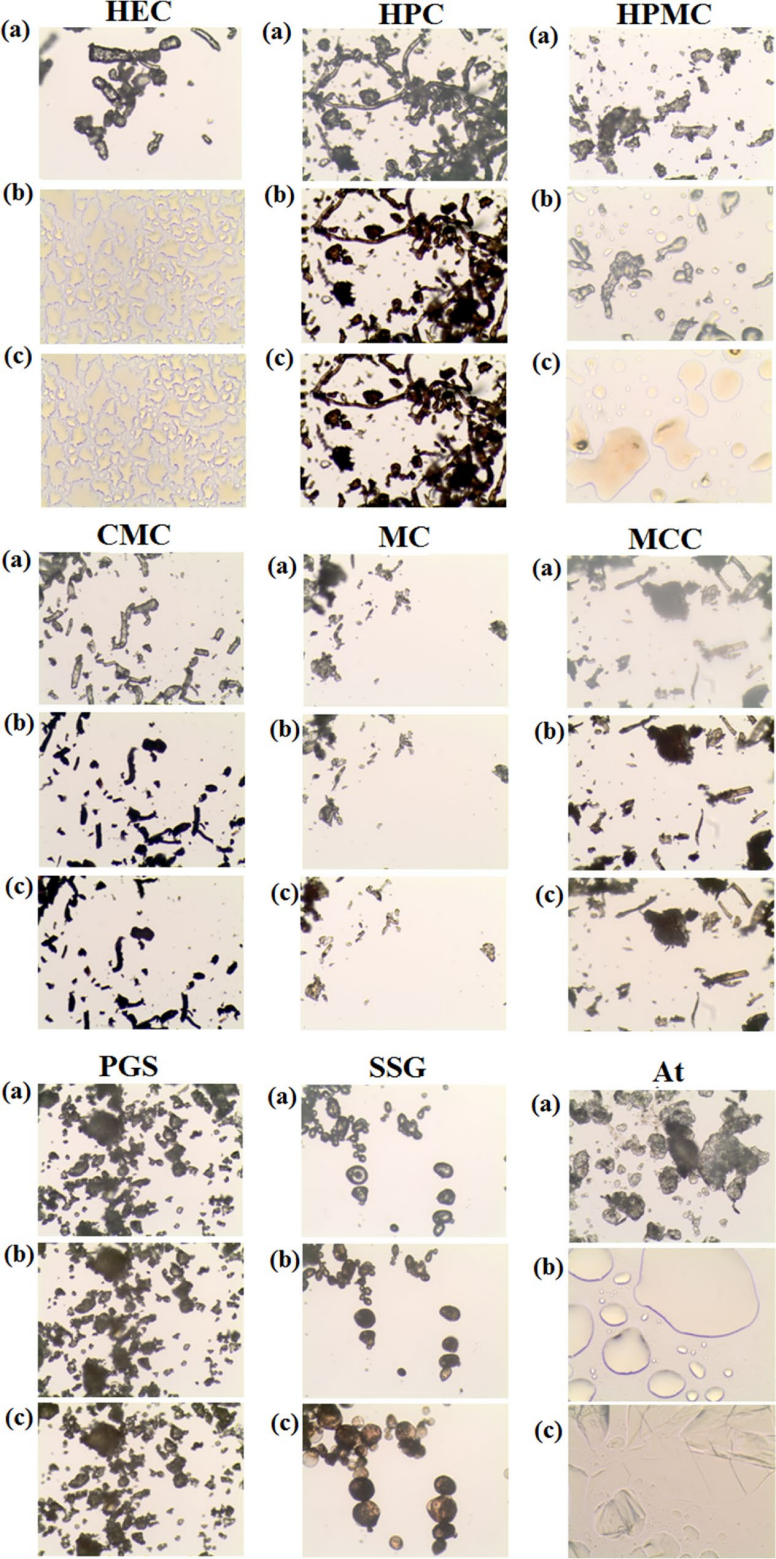
HSM micrograph for hydroxyethylcellulose (HEC), hydroxypropylcellulose (HPC), hydroxypropylmethylcellulose (HPMC), sodium carboxymethylcellulose (CMC), methylcellulose (MC), microcrystalline cellulose (MCC), pregelatinized starch (PGS), and sodium starch glycolate (SSG). Measurements were performed at **a** 25 °C, **b**, 250 °C for polymers and 155 °C for atenolol, and **c** after cooling to 25 °C

HSM of polysaccharides (Fig. 4) showed that HPMC undergoes glass transition at about 200 °C, followed by decomposition, confirmed by browning, whereas in contrast, HEC softens at about 200 °C. These data are consistent with the literature [17]. With the exception of SSG, the excipients are stable up to about 250 °C, at which temperature they char due to decomposition. SSG decomposes at 220 °C. On the other hand, atenolol melts at about 154 °C (Fig. 4) and recrystallizes after cooling.

**Fig. 5.**
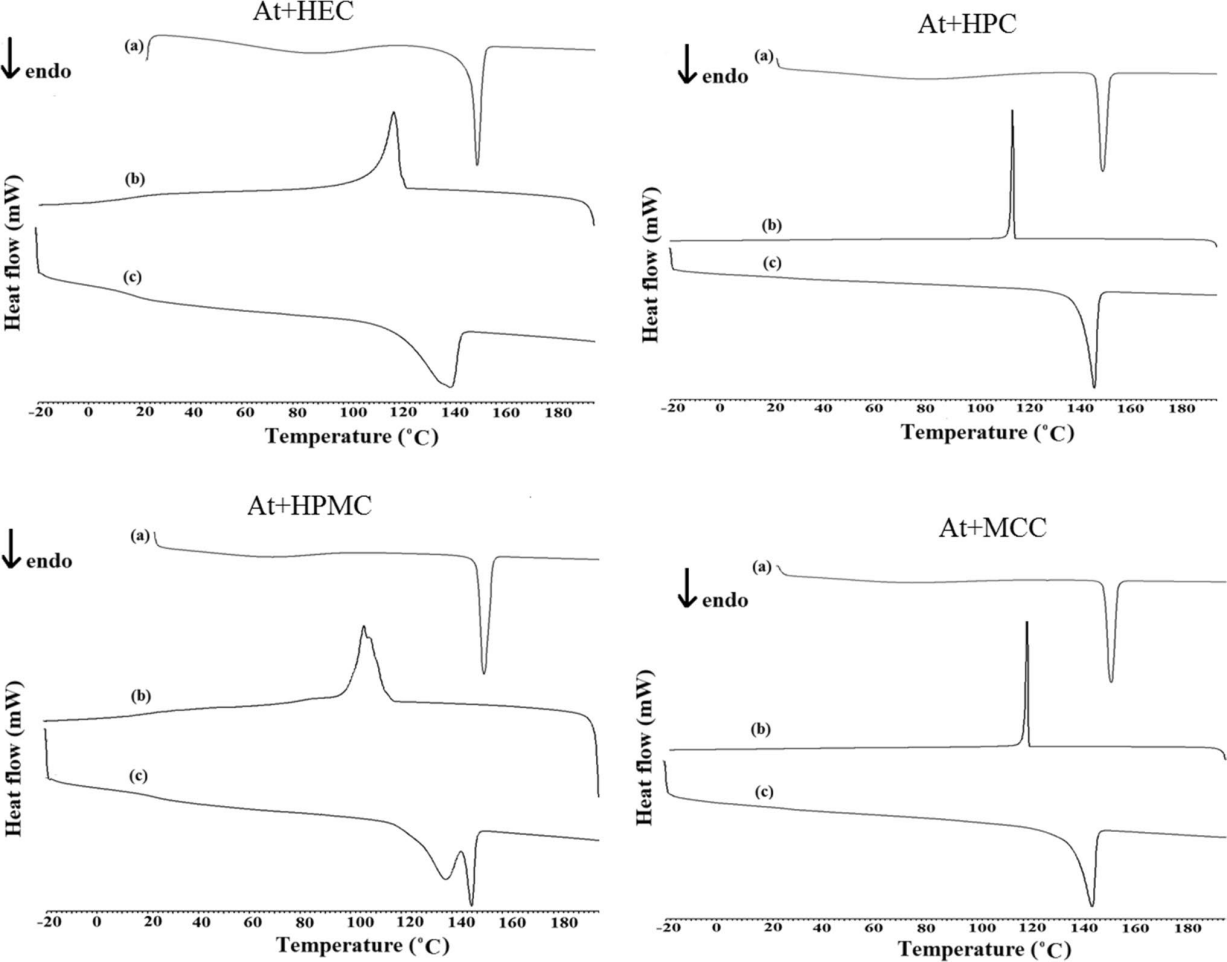
DSC curves for 50:50 m/m mixtures of atenolol with **A** HEC, **B** HPC, **C** HPMC, and **D** MCC, where **a** heating, **b** cooling, and **c** reheating

**Fig. 6.**
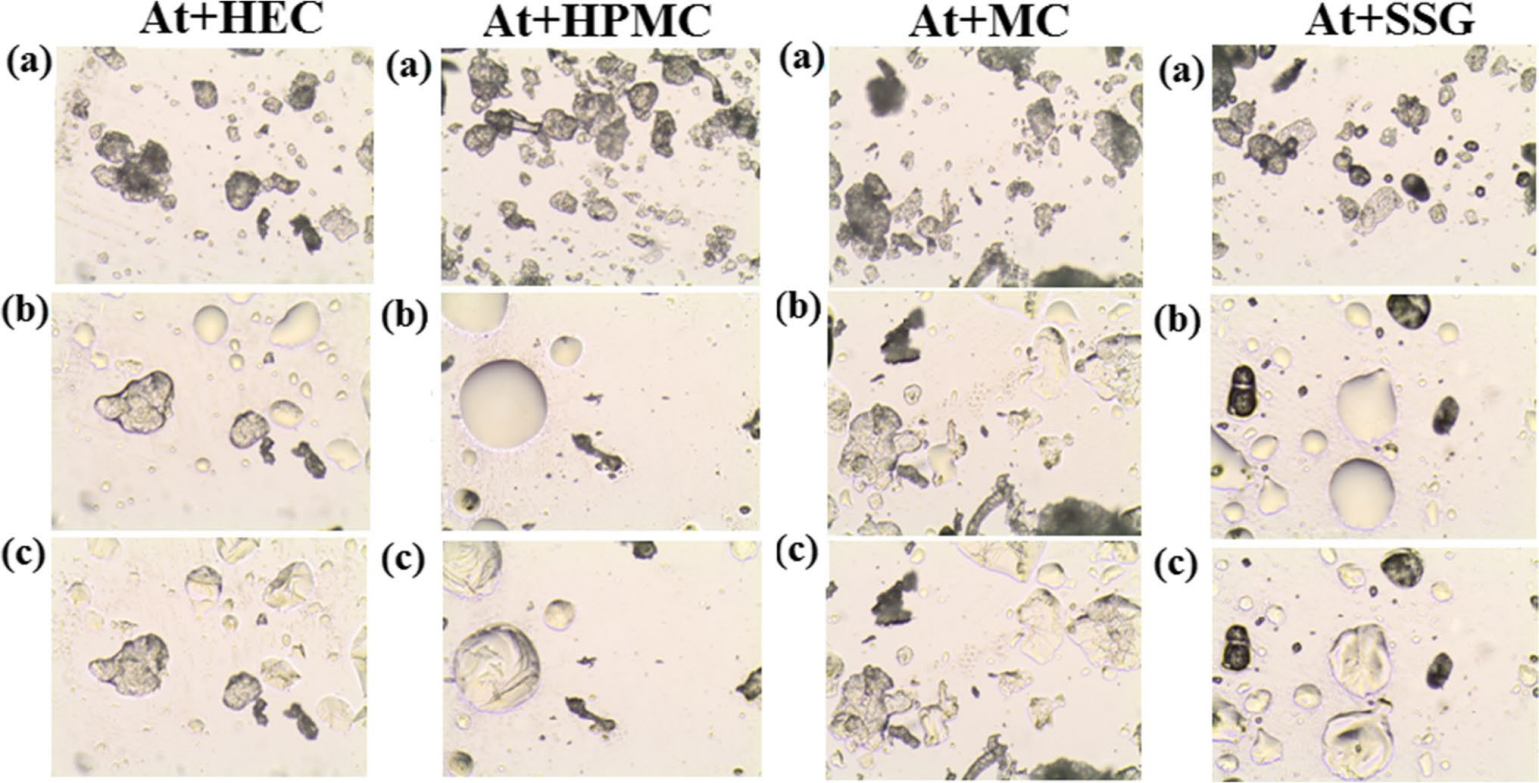
HSM micrograph for selected binary mixtures of atenolol (At) with polymeric excipients: HEC, HPMC, MC, and SSG. Measurements were performed at **a** 25 °C, **b** 155 °C, and **c** after cooling to 25 °C

DSC cooling curve of atenolol shows recrystallization at 122.4 °C. On reheating, atenolol melts at 151.4 °C. In the case of atenolol mixtures with selected polymeric excipients (Fig. 5), the melting peak of atenolol and its recrystallization while cooling and melting after reheating were all observed. However, as the mixture of atenolol with HEC (Fig. 5) recrystallizes, the DSC peak of atenolol has a different shape and is widened and fuzzy. A similar situation occurs for atenolol mixture and HPMC (Fig. 5), where two overlapping exothermic peaks are observed during the cooling process and two successive endothermic effects on reheating. Hence, incompatibility may be implied in the atenolol mixtures with HEC and HPMC. The temperatures of melting and recrystallization and the heats of fusion of atenolol alone and in the mixtures are presented in Table III. These data revealed that during the first heating of atenolol mixtures with polymeric excipients, only slight shifts of the onset and peak temperatures of atenolol towards lower values were observed due to the mixing process. The enthalpy values for the mixtures were about half lower than the enthalpy of atenolol alone, reflecting the 50% content of atenolol in the mixtures. Slightly lower values of the enthalpy of fusion of atenolol were obtained for mixtures with HEC, HPMC, and MC. In turn, during cooling, the lowest values of enthalpy are due to crystallization of atenolol in mixtures with HEC (25.56 J/g), HPMC (30.03 J/g), and MC (30.88 J/g). This suggests interactions between atenolol and HEC, HPMC, and MC. Furthermore, during the second heating, the lowest enthalpy values were recorded for atenolol in mixtures with HEC and HPMC. This is due to fact that contamination created during slight thermal degradation of polymeric excipients affect the enthalpy change of drug substance [18–21]. The values of the enthalpy of melting for atenolol in these mixtures indicate interactions between atenolol and HEC, HPMC, or MC. HSM study (Fig. 6) also revealed that atenolol in mixtures with the remaining polysaccharides melts at about 154 °C and recrystallizes during cooling. The curves for the first heating of At-HEC and At-HPMC mixtures showed that the melting point of atenolol is slightly shifted towards the lower values as compared to that of atenolol alone (in At-HEC mixture melting point is shifted from 154.4 °C to 153.1 °C and in At-HPMC mixture to 153.7 °C). This means that crystalline atenolol has melted in these mixtures. In contrast, the cooling curve of the At-HEC mixture showed the widened peak of recrystallization of atenolol at about 121.3°C (pure atenolol recrystallizes at 122.4°C). This may indicate the partial dissolution of atenolol in HEC. In contrast to At-HEC mixture, the cooling curve of At-HPMC mixture shows widened peak which is shifted more than 10°C towards the lower temperature, at ~ 105 °C. It can indicate significant dissolution of atenolol in HPMC. In turn, the reheating curve of At-HEC mixture contains a broadened peak shifted to 140.5 °C compared to the original peak of atenolol remelting at 151.4 °C. This indicates the presence of some more crystalline atenolol. The reheating curve for At-HPMC mixture shows two peaks at 138.3°C and at 148.4°C. This indicate the presence of atenolol, which is somewhat crystalline.

**Table III:** Results of DSC for Atenolol and Mixtures with Polymeric Excipients

API/mixture	DSC heating			DSC cooling			DSC reheating		
	*T*_onset_ (°C)	*T*_peak_ (°C)	Enthalpy (J/g)	*T*_onset_ (°C)	*T*_peak_ (°C)	Enthalpy (J/g)	*T*_onset_ (°C)	*T*_peak_ (°C)	Enthalpy (J/g)
Atenolol	152.60	154.36	− 137.13	121.02	122.39	102.47	146.31	151.31	− 108.35
Mixture with HEC	151.40	153.11	− 56.98	124.91	121.32	25.56	123.38	140.49	− 31.02
Mixture with HPC	151.88	153.71	− 71.72	117.77	117.90	40.15	144.16	149.13	− 44.22
Mixture with HPMC	151.20	153.69	− 65.70	109.38	104.99	30.03	128.08	138.32 148.45	− 35.76
Mixture with CMC	151.52	153.49	− 68.84	121.85	122.10	48.40	145.41	149.41	− 52.37
Mixture with MC	151.82	154.02	− 65.92	119.19	117.42	30.88	145.72	149.83	− 41.31
Mixture with MCC	151.86	154.01	− 74.08	119.88	120.60	48.16	143.06	148.62	− 54.32
Mixture with PGS	152.16	154.15	− 66.78	125.77	125.84	48.28	145.95	150.23	− 53.01
Mixture with SSG	151.62	153.48	− 69.35	120.37	121.31	49.37	145.67	149.85	− 55.08

**Fig. 7.**
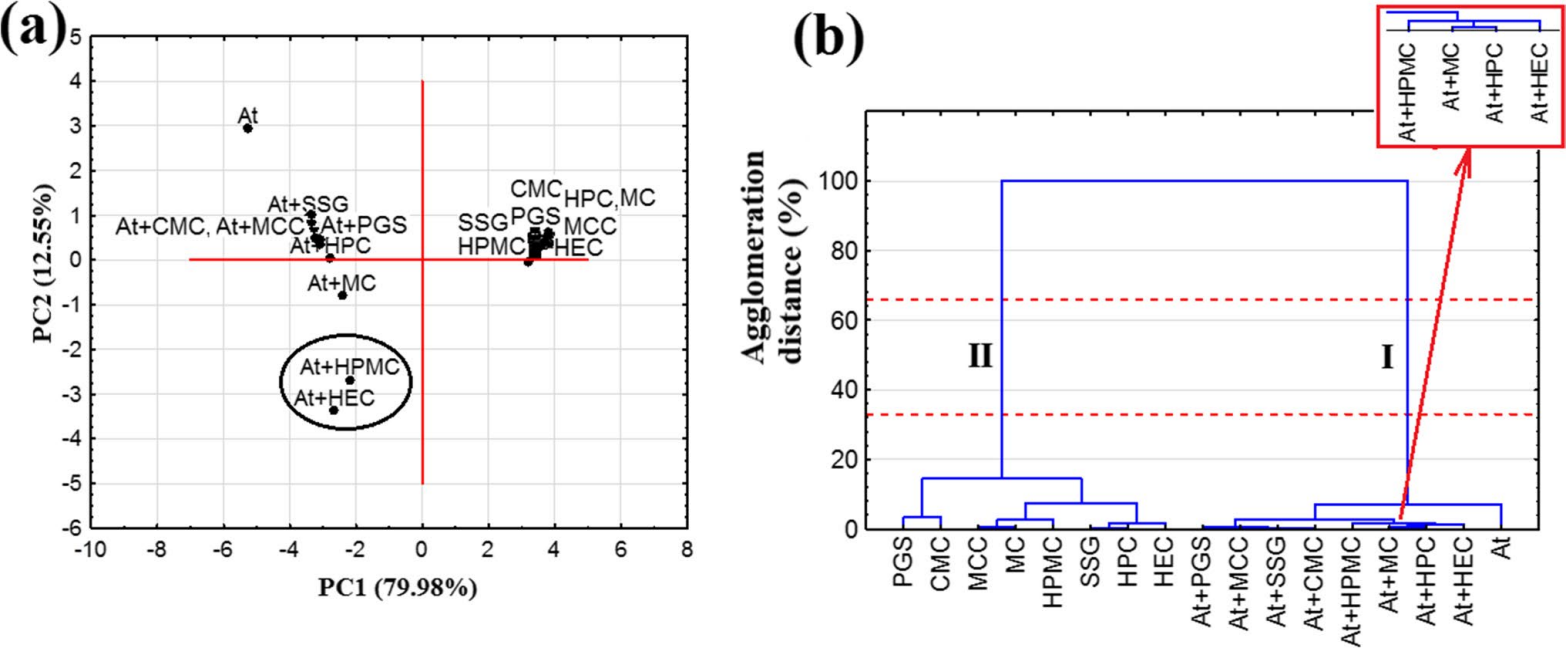
**a** PCA score scatterplot and **b** HCA dendrogram determined using the data acquired from the DSC curves

PCA was used to verify the findings acquired from DSC. Since the first and second principal components (PC1 and PC2) explain more than 92% of the total variance, the results of PCA calculations can be plotted using a two-dimensional score scatterplot (Fig. 7a). Moreover, since subsequent PCs explain the lower percent of the total variance, for example, PC3, 3.10%; PC4, 2.29%; PC5, 1.66%; and PC6, 0.19%, they were not taken into consideration. As can be seen from Fig. 7a, all polymeric excipients are grouped on the right-hand section of the PCA plot at positive values for PC1 and PC2. This indicates a significant similarity between these substances. Atenolol mixtures with HPMC and HEC (At + HPMC, At + HEC) form a cluster on the left part of the plot at negative PC1 and PC2 values. The remaining mixtures and atenolol can also be found to the left but at positive PC2 values, while the atenolol mixture with MC (At + MC) is located at a slightly negative PC2 value. Such distribution of the samples, excluding HPMC and HEC mixtures, suggests a similarity between the DSC curves of the mixtures and atenolol, implying that the ingredients are compatible. PC1 (which explains about 80% of the total variance) distinguishes between the mixtures of At + HPC, At + CMC, At + MC, At + MCC, At + PGS, and At + SSG (DSC peak of atenolol was unchanged) and the mixtures of At + HEC and At + HPMC (DSC peak of atenolol was changed).

HCA calculations for the DSC data indicate a tendency for two clusters to form between 33 and 66% of the maximum distance (Fig. 7b). The second of these is created by all polymeric excipients and demonstrates the similarity between the DSC curves of these substances. The first is formed by atenolol and its mixtures with all polymeric excipients. The grouping of the samples together in one cluster suggests a similarity of DSC curves, i.e., that all polymeric excipients are compatible with API. In the case of incompatible mixtures (atenolol mixtures with HEC and HPMC), it can be seen that they form a common cluster at one of the lowest agglomeration distance. Within this cluster is a separate cluster composed of atenolol mixtures with MC and HPC (Fig. 7b). This localization of samples is also reflected in the PCA and the distribution of samples along PC2. Atenolol mixtures with HPMC and HEC (Fig. 7a) are close to each other and stand out from the remaining samples. Near these two mixtures can be found the atenolol mixture with MC, next to which is the mixture with HPC.

### FTIR as a Supporting Technique

**Fig. 8.**
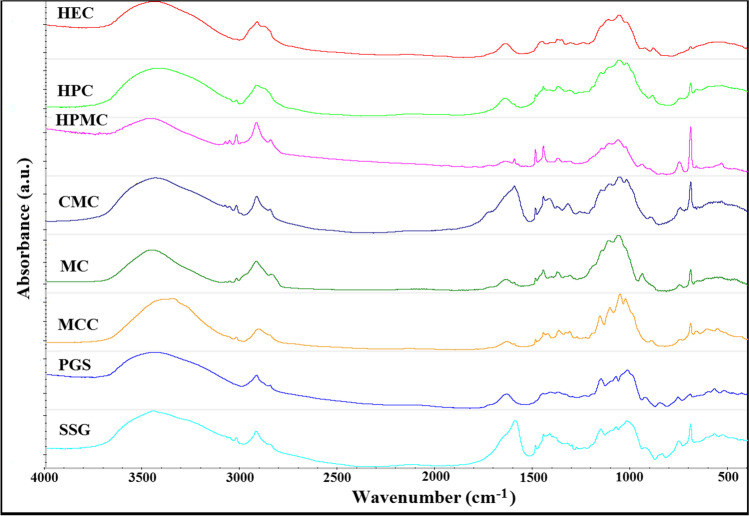
FTIR spectra of polymeric excipients

FTIR is used as a tool to support the detection of incompatibility displayed by DSC. Polysaccharides absorb IR radiation at similar frequency ranges (Fig. 8) due to the presence of the same chemical groups in their structures, and these regions are characteristic of all carbohydrates [22, 23]. There are spectral ranges for (1) O–H and C–H stretching vibrations at 3600–2800 cm^−1^; (2) HC–H and CH_2_–OH vibrations in the range of 1500–1200 cm^−1^; (3) the area of stretching bands of CO at 1200–950 cm^−1^; (4) deformation region of C–OH, C–CH, and O–CH group at 950–700 cm^−1^; (5) exocyclic deformation vibrations (CCO) in the range of 700–500 cm^−1^; and (6) endocyclic deformation vibrations (CCO, CCC) below 500 cm^−1^.

**Fig. 9.**
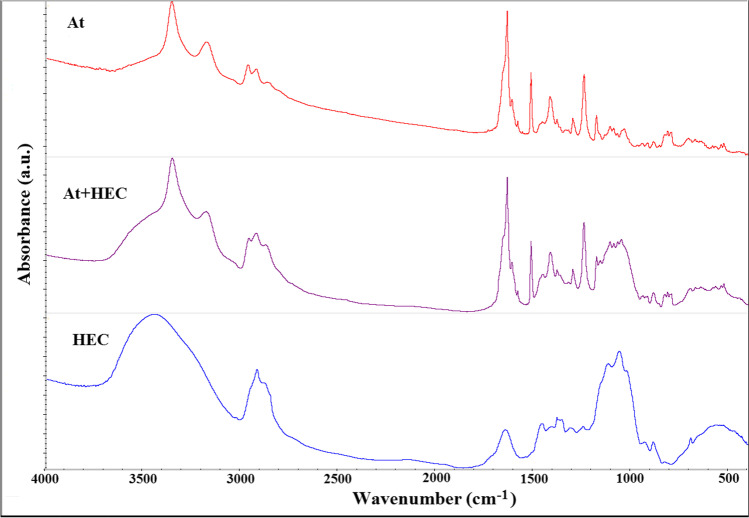
FTIR spectra for atenolol (At), hydroxyethylcellulose (HEC), and their mixture (At + HEC)

The FTIR spectrum of atenolol (Fig. 9) shows absorption bands characteristic for amides — intense stretching vibrations of the N–H groups at 3350 cm^−1^ and 3180 cm^−1^, and also for secondary amines, N–H stretching vibrations in the spectral range of 3350–3310 cm^−1^. Also observable are the stretching vibration bands of the O–H group at 3550–3200 cm^−1^ and O–H bending vibration at 1420–1330 cm^−1^ and C–H group vibration bands at 3000–2800 cm^−1^. The absorption band at 1650 cm^−1^ is attributed to the stretching vibrations of the H_2_N–C = O group of primary amides [24].

**Fig. 10.**
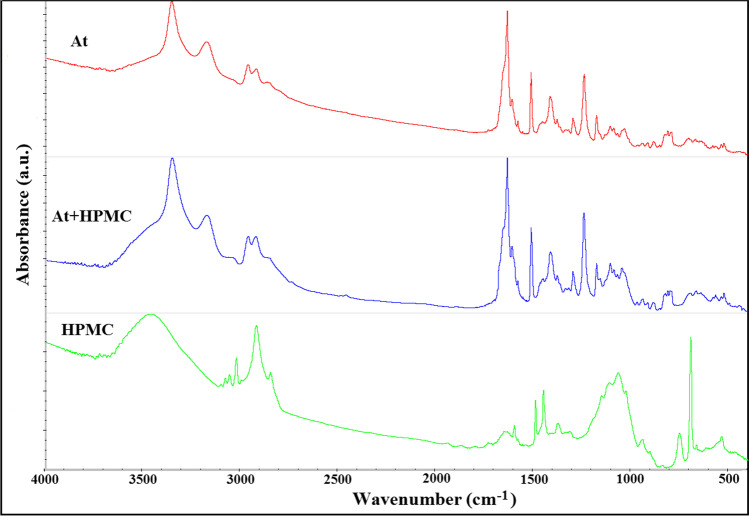
FTIR spectra for atenolol (At), hydroxypropylmethylcellulose (HPMC), and their mixture (At + HPMC)

FTIR spectrum of physical mixture was compared with the spectra of individual ingredients. Appearance or disappearance of absorption band(s) of ingredients, broadening characteristic band(s) or alteration in intensity of band(s) in the spectra of the mixtures indicate incompatibilities between ingredients [25]. In the case of atenolol spectrum, the most characteristic changes in the absorption bands due to incompatibility were observed in the spectral range of 3600 – 3000 cm^–1^ and 1700 – 1330 cm^-1^, which is related to the functional groups CONH_2_, NH and OH that can form hydrogen bonds. In this spectral range there are – the N-H groups vibrations at 3350 - 3180 cm^-1^ and the O-H group vibrations at 3550 – 3200 cm^-1^, the H_2_N-C=O group vibration at 1650 cm^-1^ and the O-H group vibrations at 1420 – 1330 cm^-1^. These parts of the FTIR data illustrate incompatibility between atenolol and polymeric excipients. FTIR spectrum of atenolol mixture with HEC (Fig. 9) showed overlapping bands from the two ingredients in the ranges of 3500–2800 cm^−1^ and 1700–500 cm^−1^. In the spectrum of atenolol mixture with HPMC (Fig. 10), changes with respect to HPMC were observed, and some bands of atenolol and HPMC overlapped. The spectra of atenolol and HPMC contain bands of chemical groups in similar spectral ranges.

**Fig. 11.**
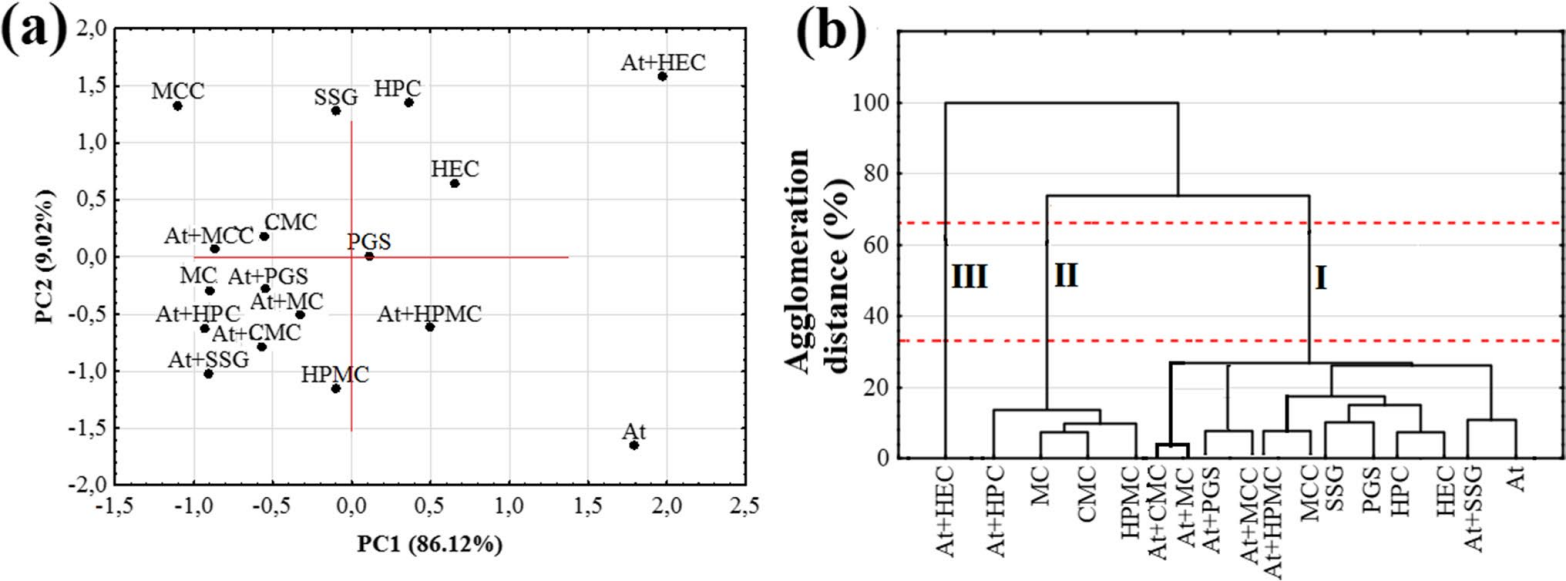
**a** PCA score scatterplot and **b** HCA dendrogram determined using the data acquired from the FTIR spectra

PCA calculations based on the FTIR data for atenolol, polymeric excipients, and their binary mixtures revealed that the two first principal components (PC1 and PC2) account for more than 95% of the total variance. On the PCA plot (Fig. 11a), atenolol and its mixture with HEC are located on the right-hand side, atenolol at a positive value for PC1, and a negative for PC2, whereas its mixture has a positive value for both PC1 and PC2. In contrast, atenolol mixture with HPMC is distant from the atenolol, along with decreasing PC1 values and increasing negative PC2 values. On the other hand, HPMC can be found at negative values for PC2 and slightly negative PC1 values. HEC and HPC are located at positive PC1 and PC2 values. The remaining mixtures and polymeric excipients are grouped on the left-hand side of the plot at PC1 negative and PC2 negative and positive values. Hence, PCA shows differences in the localization of atenolol on the plot versus polymeric excipients and their mixtures. This is related to the different appearance of FTIR absorption bands of atenolol, polymeric excipients, and their mixtures. Moreover, such localization of samples also indicates differences in the appearance of absorption bands of atenolol mixture with HEC in relation to those of polymeric excipients and mixtures.

HCA calculations for the FTIR data revealed three clusters above 33% of the maximum distance (Fig. 11b), the first consisting of atenolol, polymeric excipients (with the exception of MC, CMC, and HPMC), and also mixtures, excluding those with HEC and HPC. The second cluster includes atenolol mixture with HPC and three polymeric excipients such as MC, CMC, and HPMC, while the third cluster is formed solely by atenolol mixture with HEC.

According to the literature data, DSC has revealed that HPMC is incompatible with rosmarinic acid [26] and paracetamol [27]. However, FTIR did not confirm these findings. Probably, the use of multivariate statistical methods might improve the interpretation of the FTIR spectra, enabling the detection of incompatibility. Veras et al. [26] suggest that high temperatures may be responsible for the incompatibility between HPMC and rosmarinic acid, whereas interactions of HPMC with paracetamol [27] have manifested as decreased paracetamol crystallinity.

The detection of the incompatibility between HPMC and atenolol depends on polymer physicochemical properties. HPMC, for example, has the ability to swell and form a gel layer, which allows control of the drug release rate on the surface of matrix systems, and therefore, it is used in the development of controlled release dosage forms [28–30]. Additionally, HPMC has favorable mucoadhesive properties. The mechanism of this phenomenon for non-ionic HPMC is the interpenetration of HPMC molecules with mucin chains and the formation of hydrogen bonds [28]. Therefore, the incompatibility of atenolol with HPMC detected by DSC may be due to the polymer forming a viscous gel layer.

In the case of HEC, Pires Maximiano et al. [31] have used DSC to show its incompatibility with benznidazole, while FTIR did not show any significant changes in the spectra of the mixture. Although HEC has mucoadhesive properties [32] similar to HPMC, it seems in this case that the main mucoadhesion mechanism is physical penetration and subsequent entanglement of polymer chains.

**Fig. 12.**
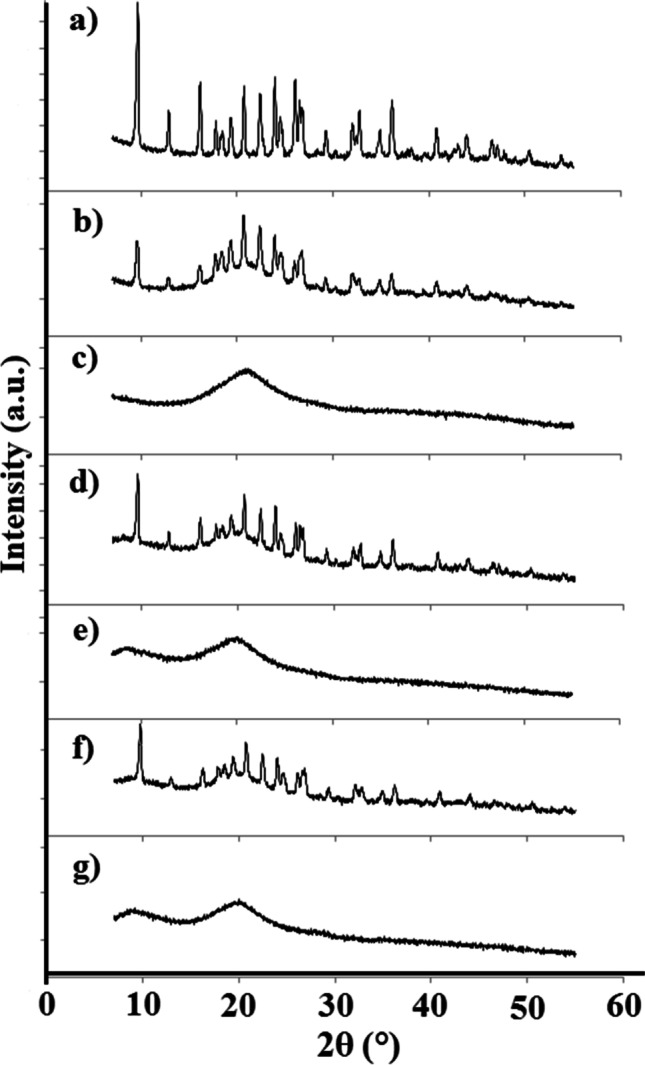
PXRD patterns at ambient temperature for **a** atenolol, **b** At-HEC mixture, **c** HEC, **d** At-HPMC mixture, **e** HPMC, **f** At-MC mixture, and **g** MC

**Fig. 13.**
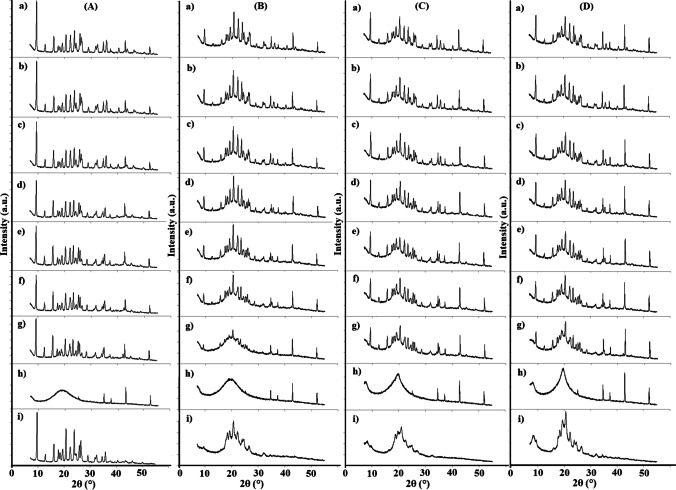
PXRD patterns for **A** atenolol, **B** At-HEC mixture, **C** At-HPMC mixture, and **D** At-MC mixture at temperature of **a** 25 °C, **b** 65 °C, **c** 85 °C, **d** 105 °C, **e** 125 °C, **f** 135 °C, **g** 145 °C, **h** 155 °C, and **i** after cooling to 25 °C

PXRD patterns of atenolol, HEC, HPMC, MC, and their mixtures are presented in Fig. 12 (room temperature) and Fig. 13 (different temperatures). The diffraction pattern of atenolol at room temperature (Fig. 12a) shows the most intense line at 2θ of 9.51 and other secondary lines at 2θ of 12.72, 15.96, 17.55, 17.97, 19.22, 20.52, 22.22, 23.73, 23.73, 25.82, 26.31, 29.08, 31.73, 32.40, and 35.76, revealing the crystalline nature of atenolol. Diffraction patterns of the excipients (Fig. 12c, e, g) also show one broad peak that can be discerned with many undefined diffused low-intensity peaks, indicating the amorphous state of the excipients. The PXRD measurements for atenolol at increasing temperature values show a gradual reduction of the crystalline form of atenolol (Fig. 13A, a–g), while at 154 °C, atenolol melted (Fig. 13A, h), as evidenced by the amorphous form. However, after cooling to room temperature (Fig. 13A, i), atenolol recrystallizes.

**Fig. 14.**
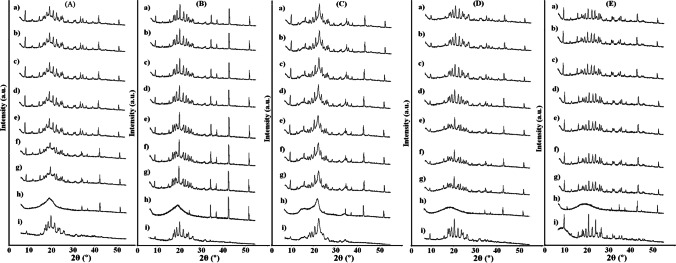
PXRD patterns for (**A**) At-HPC mixture, (**B**) At-CMC mixture, (**C**) At-MCC mixture, (**D**) At-PGS mixture, and (**E**) At-SSG mixture at temperature of (**a**) 25 °C, (**b**) 65 °C, (**c**) 85 °C, (**d**) 105 °C, (**e**) 125 °C, (**f**) 135 °C, (**g**) 145 °C, (**h**) 155 °C, and (**i**) after cooling to 25 °C

In order to determine the structural changes that may occur when mixing atenolol with the excipient, PXRD pattern of the binary mixture was compared with ones of the individual ingredients. PXRD patterns of binary mixtures of atenolol with HEC, HPMC, and MC at room temperature (Fig. 12b, d, f) showed the sum of the patterns of ingredients with no changes in the positions of atenolol lines. This indicates that the ingredients are compatible when mixed at room temperature. Comparing PXRD patterns of atenolol (Fig. 13A) and its mixtures, it was revealed that the At-HEC mixture in the temperature range of 25–145 °C (Fig. 13B, a–g) showed a significant reduction in the intensity of the highest line of atenolol at 2θ value of 9.51. Moreover, the pattern of this mixture after cooling from 155 °C to room temperature (Fig. 13B, i) clearly shows the reduction of the intensity of all diffraction lines of atenolol, indicating the reduction of atenolol crystallinity. For atenolol mixtures with HPMC and MC, a reduction in the crystallinity of atenolol (Fig. 13C, i, and D, i) is also observed in PXRD patterns after cooling these mixtures to room temperature. In contrast to PXRD patterns of the mixtures, the pattern of atenolol after cooling to 25 °C (Fig. 13A, i) exhibits all the diffraction lines with the same intensity as atenolol analyzed at room temperature. This indicates the occurrence of incompatibility between atenolol and polymeric excipients (HEC, HPMC, and MC), the cause of which may be the formation of solid dispersion systems [33]. This is consistent with the literature data, since reduction in crystallinity is recognized as a physical incompatibility. The physical properties of the drug substance undergo change, for example, its solubility, dissolution rate, stability [34]. In turn, Fig. 14 shows the diffraction patterns for compatible mixtures. In these mixtures, the crystallinity of atenolol was slightly reduced. The methods used in our study have shown that both thermal and non-thermal methods are useful in the study of incompatibility. The heating-cooling-reheating test showed changes in the melting point, temperature of crystallization and remelting point of atenolol, and changes in shape and width of peaks for At-HEC and At-HPMC mixtures, which were confirmed by the formation of PCA and HCA clusters in which these two mixtures were grouped, unlike to the other mixtures. Moreover, no significant changes were found in the FTIR spectra of these mixtures, although the bands of ingredients probably overlapped. The lack of the appearance of new bands or the lack of disappearance of the bands of ingredients exclude chemical interactions at room temperature. Also, PXRD at room temperature did not show changes in crystalline nature of atenolol, but while heating, a decrease in crystallinity of atenolol was observed. Changes in atenolol in the presence of HEC or HPMC indicate physical incompatibility, but these are favorable since they increase the solubility of atenolol.

## CONCLUSIONS

The heating–cooling–reheating program is useful for detecting potential incompatibilities between atenolol and polymeric excipients, as it permits a clear definition of the incompatibility in comparison to the standard DSC test carried out only in the heating cycle. Studies allow the phase transitions which ingredients undergo in the mixture to be observed, and the addition of cooling and reheating cycles to standard heating revealed changes in atenolol phase transitions in the presence of polymeric excipients which could not be seen with standard heating. The PCA used to interpret the DSC data unambiguously revealed that in the case of atenolol mixtures with HEC, HPMC, and MC, the melting peak of drug substance changes during cooling and reheating, indicating the influence of these polymeric excipients on the properties of atenolol.

HSM is a supportive tool for DSC and can serve as a green and less expensive compatibility test approach. The use of HSM is beneficial because it enables visual observation of transitions in the sample during the heating process. On the other hand, FTIR does not show changes in atenolol absorption bands after mixing with polymeric excipients. Thus, without the use of multivariate statistical methods, FTIR does not confirm the outcomes of DSC. As a result of the present research, incompatibility was found in mixtures of atenolol with two polymers: HEC, HPMC, and MC. PXRD measurements at room temperature revealed that the crystallinity of atenolol did not change in the mixtures with HEC, HPMC, and MC. However, its crystallinity was reduced in the mixtures previously heated up to 155 °C and then cooled to 25 °C.
